# Abstracts from Foreign Journals

**Published:** 1903-01

**Authors:** S. J. J. Harger

**Affiliations:** Philadelphia, Pa.


### ABSTRACTS FROM FOREIGN JOURNALS.

### FRENCH.

UNDER THE DIRECTION OF S. J. J. HARGER, V.M.D.,
PHILADELPHIA, PA.

TREATMENT OF FISTULA OF THE URACHUS. The wall of the urachus is attached to the border of the umbilical opening. This fact predisposes the orifice to remain open when the cord is torn off too short or sometimes when the stump has sloughed off after ligaturing. The urine will continue to escape drop by drop, and complications of pyæmia and polyarthritis will follow.

Various cicatrizing and caustic applications, tincture of iodine, as well as resection of the urachus have often given poor results. Galvisberg employed the following treatment with remarkable results: The region is shaved and disinfected; a 15 per cent. solution of common salt, to which are added a few drops of phenic acid, is injected subcutaneously at several points around the umbilical orifice; the surface is then covered with iodoform collodion. In several hours a diffuse swelling forms which closes the urachal opening, and the dropping of urine ceases.

The author has found this trouble more common in females than in males, but does not venture whether, like umbilical hernia, it is hereditary.—*Ann. de Med. Vet.*

CONTRIBUTION TO THE STUDY OF ANEURISM IN THE HORSE. M. Sticker describes the formation of verminous aneurism as follows: The larvæ, carried by the blood, are arrested in the vasa vasorum of the large arterial branches of the posterior aorta and excite hypertrophic inflammation of the middle tunic of the vessel. These facts have been demonstrated by making sections of recent aneurisms. As the larvæ grow they form galleries in the middle tunic and penetrate toward the internal tunic, which in turn becomes inflamed; it is only at this moment that the endarteritis develops, followed by the formation of a thrombus.

The larva does not possess the attaching apparatus of the adult worm and cannot fasten itself to the wall of the large vessel while in the circulation. In old aneurisms in which the galleries already intersect the internal tunic one or both extremities of the larva may be seen protruding into the lumen of the artery. Exceptionally the parasite may be found free in the blood, but this is seen only in old aneurisms when the galleries have opened into the lumen of the vessel.

Sticker calls attention to the fact that his observations upon this point are in contradiction to the idea of many other authors who consider the endarteritis and thrombosis as the primary lesion and the meso-arteritis, at first acute and afterward chronic, with hyperplasia of the external tunic, secondary.—*Ann. de Med. Vet.*

Production of Sugar by the Muscles. Cadeac and Maignon have found sugar in muscles crushed or constricted by ligature. Afterward they undertook to demonstrate whether the sugar is deposited at the traumatic centre or elaborated in the muscles themselves.

The following are their conclusions:

1. The muscles, like the liver, always produce sugar after death.
2. Muscles plunged in oil at 37° C. will produce more sugar than at the same temperature in the atmosphere.
3. Muscles surrounded by ice produce the least sugar.
4. Muscles crushed or compressed elaborate the maximum quantity of sugar.
5. This function of muscles is independent of all putrefaction.—*Ann. de Med. Vet.*

Preventive Treatment of Rabies in the Horse. Galtier, Nocard, and Roux demonstrated that saliva of rabic animals introduced into the veins of the horse and sheep does not cause rabies, but seems to confer immunity. They demonstrated, besides, that the intravenous injection of pure rabic virus (medulla) in horse, ox, and sheep twenty-four hours after the intra-ocular inoculation of a virulent cord is sufficient to prevent the evolution of rabies.

Monet first applied this principle practically to three cows that had been bitten by a rabid dog, each of which received 5 cc. of an emulsion of the medulla of the dog, eighty-four, seventy-nine, and one hundred and eighteen days after the bite was inflicted. None of these animals became rabid.

Rabieaux injected 16 c.c. of an emulsion of the brain of a rabid dog into the veins of an ass two days after it was bitten by the dog. The animal died.

Conet, in 1898, tried to immunize five horses bitten by a rabid dog. One injection of 5 c.c. of the medulla of the dog was made into the jugular vein; the injection was repeated in twenty-four hours.

The first horse was treated five days after the bite was inflicted; he died six months afterward after showing symptoms of paraplegia.

The second was treated seven days after the accident, and died in seventy-nine days.

The third, treated after four and a half days, died in two hundred days.

The fourth was sold after seventy-one days without showing any symptoms. The fifth died from rabies after one hundred and eighty days.

Of these five horses, three died from rabies. The cause of the paraplegia in the first is not well understood, and in the fourth the result was unknown.

The question to be raised from these observations is whether immunity would have been obtained had the injection been made immediately after the horses were bitten, or had the animals received successive injections at intervals. The treatment seems to have very much lengthened the period of incubation.—*Ann. de Med. Vet.*

PSEUDOTUBERCULOSIS IN THE DOG. Ducourneau and Jayles report the following case: An English bull dog, three months old, had been affected with distemper of the nasobronchial form, but his convalescence was slow and the general health bad. Among the symptoms were enlarged abdomen, polydipsia, polyuria, absence of sugar and of albumin in the urine, no appetite. The patient was suspected of tuberculosis, but tuberculinization was negative. In spite of all treatment the general state became aggravated, the ascites increased, diarrhœa became abundant, and the dog was sacrificed.

*Autopsy.* The liver was of enormous volume, of a reddish-brown color, marbled, with numerous yellowish-white spots, and bathed in eleven litres of peritoneal fluid. It weighed two kilos (410), or one-eighth of the body-weight; its surface was roughened by numerous tubercles without any caseous centres, and varying in dimensions from 1 to 22 mm. These tubercles existed throughout the thickness of the liver, where they had the same characters; some were also found upon the pancreas, great omentum, superior border of the left lung, and on the left auricle.

A histological examination of the liver, made by Professor Bimes, demonstrated that the lesions of this organ were not of a tuberculous nature; it was a question of intravascular pseudotubercles developed in consequence of a microbic infection of intestinal origin, but of an undetermined nature.—*Rec. de Med. Vet.*

DIAPHRAGMATIC HERNIA IN THE DOG. Clinically the patient showed intense dyspnœa for three days.

*Post-mortem* The pericardium was greatly dilated, as in pericarditis, with much exudation; but, instead of the latter, the pericardial sac was filled with a mass of intestinal (small) convolutions surrounding and compressing the heart. The pericardium at its apex, where it is attached to the diaphragm, had an opening corresponding at the same point to an opening in the diaphragm whose development had at a certain moment become arrested.

The hernia was recent, since the intestine, not even at the level of the diaphragmatic ring, did not show any signs of strangulation.

A peculiar anomaly of the liver predisposed to the displacement of the intestine. One of its lobes situated on the median line was absent, and the right and left parts of the liver were united only by a piece of hepatic tissue as large as a crayon. At a given moment the intestine passed between these two parts of the liver and penetrated the diaphragmatic opening.

This anomaly of the pericardium and diaphragm shows a loss of substance and was congenital; there was no evidence of rupture or inflammation of any part. This is a very rare anomaly.—*Rec. de Med. Vet.*

Cancer of Bronchial Origin in the Dog. Small masses of epithelium covering the bronchia or gills in the fœtus may be the point of development of cancer. These are more frequent in man, but a few have been observed by Petit in the dog. They illustrate Cohnheim's theory of the causation of tumors. According to this theory small masses of embryonic cells remain embedded in the tissues in a latent form; from some obscure cause these cells commence to proliferate and develop into a characteristic growth. Cancer of the jaw develops from the paradental epithelial débris embedded in the gums.

Cancers of bronchial origin may be clinically confounded with goitre. Like the latter, they are situated in the region of the throat, where they form on the right or the left side a more or less pronounced enlargement. They are quite hard, fastened to the surrounding tissues, and covered by the skin, healthy and but little adherent; the neighboring lymphatic glands are invaded, small nodules may be found along the course of the carotid artery, and metastatic growths may develop in the internal organs. Other symptoms may result from general cachexia or compression of important nerves and bloodvessels in the region of the neck.

Dissection of the tumor proves the integrity of the thyroids, parotid, and maxillary salivary glands, as well as the nasal, buccal, pharyngeal, and laryngeal mucous membrane. The tumor is not of cutaneous origin, because the skin is healthy and little adherent. —*Rec. de Med. Vet.*

Immunization against Dourine. Buffard and Schneider inoculated two asses with the virulent blood. Three months afterward there were no apparent signs of mal de coit and the trypanosoma could not be found in the blood. Inoculated subsequently three times at intervals of fifteen days with a considerable dose of the parasite, these animals did not manifest any reactive signs.

Three dogs, having recovered from the first attack, were reinoculated and contracted a mild form of dourine. A third inoculation remained without effect upon one of them, while the other two showed a slight reaction; since then all attempts at reinfection were negative.

The authors conclude that a first attack confers a veritable immunity, even in the dog, in which it is acquired with greater difficulty; but more prolonged observation shows that immunity is not actually realized. In the asses the disease appeared again without inoculation some months afterward.

It would appear that the trypanosoma of dourine may exist in the system for an indefinite period, due to granulations which it emits, and which play the rôle of veritable spores. The idea of latent parasitism is therefore applicable to dourine as in the bovine piroplasma; but, while in the piroplasma the latent parasitism creates a definite and durable immunity, in the ass the adult trypanosoma reappears and manifests itself by well-defined signs.—*Ann. de Med. Vet.*

Chondroma and Osteochondroma in the Mammary Gland of a Bitch. Petit reports the two following cases:

1. *A pure chondroma*, a flat disk-shaped tumor measuring 10 cm. in diameter. The surface is lobulated, but the structure is uniform and the salient cartilaginous masses are not separated from one another by fibrous tissue.

2. An osteoid chondroma as large as two fists. It consists of large islands of cartilaginous tissue almost entirely ossified and separated from one another by fibrous or myxomatous tissue hollowed out at diverse points by pseudocystic cavities.

The author recalls the frequency of tumors of the mammary gland of the bitch. This gland, like that of woman, is the seat of all varieties of tumors. The chondroma are generally associated with myxomatous, sarcomatous, fibrous, and even osseous tissue.—*Rec. de Med. Vet.*

---

Dr. Richard Ebbitt has returned to Grand Island, Nebraska, and again resumed practice after a year's travelling out of our country.

Dr. Thomas Trinder, of Memphis, Tenn., has removed to Vicksburg, Miss.

